# Young Adults’ Use of Mobile Food Delivery Apps and the Potential Impacts on Diet During the COVID-19 Pandemic: Mixed Methods Study

**DOI:** 10.2196/38959

**Published:** 2023-05-09

**Authors:** Xiang Cong Tham, Clare Whitton, Falk Müller-Riemenschneider, Nicholas Alexander Petrunoff

**Affiliations:** 1 Saw Swee Hock School of Public Health National University of Singapore Singapore Singapore; 2 School of Population Health Curtin University Perth Australia; 3 Digital Health Centre Berlin Institute of Health Charité – Universitätsmedizin Berlin Berlin Germany

**Keywords:** young adults, food delivery, mobile app, COVID-19, diet, sugar-sweetened beverages, mixed methods, fruits, vegetables, physical activity, mobile phone

## Abstract

**Background:**

A poor diet contributes substantially to the development of noncommunicable diseases. In Singapore, it is recommended to consume at least 2 servings of fruits and vegetables daily to reduce the risk of developing noncommunicable diseases. However, the adherence rate among young adults is low. The COVID-19 pandemic has led to frequent users of mobile food delivery apps (MFDAs) adopting unhealthy eating habits, including high consumption of sugar-sweetened beverages, making it crucial to gain a deeper understanding of the underlying factors driving their use patterns.

**Objective:**

We aimed to examine the use patterns of MFDAs among young adults during the COVID-19 pandemic; investigate the association between MFDA use and sociodemographic factors, dietary factors, and BMI; identify the underlying reasons for the observed use patterns of MFDAs among users; and compare the influences of MFDA use between frequent and infrequent users.

**Methods:**

A sequential mixed methods design was used involving a web-based survey and in-depth interviews with a subset of respondents. Poisson regression and thematic analysis were used to analyze the quantitative and qualitative data, respectively.

**Results:**

The quantitative results revealed that 41.7% (150/360) of participants reported using MFDAs frequently, defined as at least once a week. Although not substantial, the study found that frequent users were less likely to consume 2 servings of vegetables per day and more likely to drink sugar-sweetened beverages. Nineteen individuals who had participated in the quantitative component were selected for and completed the interviews. Qualitative analysis identified 4 primary themes: deliberations about other sources of meals versus meals purchased via MFDAs, convenience is vital, preference for unhealthy meals ordered from MFDAs most of the time, and cost is king. Before making any purchase, MFDA users consider all these themes at the same time, with cost being the most important influential factor. A conceptual framework based on these themes was presented. Lack of culinary skills and COVID-19 restrictions were also found to influence frequent use.

**Conclusions:**

This study suggests that interventions should focus on promoting healthy dietary patterns in young adults who frequently use MFDAs. Teaching cooking skills, especially among young male individuals, and time management skills could be useful to reduce reliance on MFDAs. This study highlights the need for public health policies that make healthy food options more affordable and accessible. Given the unintended changes in behavior during the pandemic, such as reduced physical activity, sedentary behavior, and altered eating patterns, it is essential to consider behavior change in interventions aimed at promoting healthy lifestyles among young adults who frequently use MFDAs. Further research is needed to evaluate the effectiveness of interventions during COVID-19 restrictions and assess the impact of the post–COVID-19 *new normal* on dietary patterns and physical activity levels.

## Introduction

### Background

Poor dietary habits are a leading cause of death worldwide because of noncommunicable diseases (NCDs) [1]. Empirical studies have suggested that adherence to recommended dietary practices, such as the daily consumption of 2 to 3 servings of fruits and vegetables, might decrease the risk of developing type 2 diabetes by 42%. In contrast, the consumption of 3 servings of sugar-sweetened beverages (SSBs) per day is associated with a 3-fold increased risk of developing type 2 diabetes in adults [2].

The Health Promotion Board of Singapore, a government organization promoting healthy living, recommends consuming at least 2 servings of fruits and 2 servings of vegetables per day, with water as the preferred beverage [3]. However, a Singapore study conducted in 2017 found that only 27.1% of young adults met the national fruit and vegetable intake guidelines [4]. Although data on the prevalence of SSB consumption among young adults in Singapore are currently unavailable, a national study involving 3430 Australian adults reported that 47.3% of the participants consumed SSBs, with 13.6% consuming at least one SSB per day [5]. Given that a Singaporean cohort study revealed that 67% of adults were overweight [6] based on the Asian BMI (≥23 kg/m^2^) [7], with SSBs being a considerable risk factor, the high prevalence of SSB consumption is concerning.

Young adulthood is a crucial period for NCD prevention, and weight gain during this phase is a substantial risk factor for NCD development. A trend analysis indicated that the incidence of obesity among young adults was increasing, with a UK study showing that young adults aged 18 to 24 years had a 4-fold higher risk of transitioning from normal weight to overweight or obese in 10 years than those aged 65 to 74 years [8].

The COVID-19 pandemic has presented opportunities for increased adoption of mobile food delivery apps (MFDAs) as a safe and convenient food purchase method given the strict enforcement of social distancing measures [9,10]. Unlike traditional takeout services, MFDAs involve a delivery service that eliminates the need for consumers to travel to a single location to purchase food and offer an array of food options from multiple restaurants that can be accessed from the comfort of one’s home or any location. This approach also grants consumers ample time to browse the available food options offered by the apps.

### Literature Review

Studies examining the use of MFDAs have shown varying prevalence rates and patterns across different countries. A study conducted in Western countries found that only 15% of participants reported using MFDAs at least once a week [11]. The study also found that demographic groups such as male individuals, ethnic minority populations, those with children aged <18 years, and those with higher educational status were more likely to use MFDAs, whereas use decreased with increasing age [11]. In contrast, a study conducted on a national sample of Kuwaiti internet users revealed a much higher prevalence of MFDA use, with 87.6% of consumers reporting use and 42.7% using MFDAs once a week [12]. In this study, higher use was observed among female consumers aged 20 to 30 years with a bachelor’s degree and a monthly income of KD 1500-2000 (US $4950-$6600) [12]. Furthermore, 76.4% of the consumers ordered fast food, whereas 73.6% of them purchased dinner via MFDAs in this study [12].

Other studies have also shown that MFDAs are often used to order unhealthy foods. A study conducted in Malaysia found that 77.6% of young adult users ordered unhealthy food, with 40.3% using MFDAs 1 to 3 times a month [13]. In a Singaporean cohort study, MFDA use was found to be associated with a decrease in vegetable orders by 15% and an increase in orders for barbecued or fried food and beverages by 11% and 4%, respectively, during COVID-19 lockdowns. The decrease in vegetable orders was further magnified by 21% when work from home (WFH) was implemented [14]. Despite the prevalence of unhealthy food ordering, a qualitative analysis found that practical needs such as price, quality, and reliability of service were the main reasons for using MFDAs [15]. Together, these findings highlight the need to better understand the patterns of MFDA use and the factors driving these patterns to develop targeted interventions for promoting healthy eating.

To the researchers’ knowledge, this is the first study that focuses on the use of MFDAs and its association with the healthfulness of users’ diets and explains the use patterns through qualitative interviews during the COVID-19 pandemic when restrictions were in place. This study’s research objectives were to (1) examine the use patterns of MFDAs among young adults during the COVID-19 pandemic; (2) investigate the association between MFDA use and sociodemographic factors, dietary factors, and BMI; (3) identify the underlying reasons for the observed use patterns of MFDAs among users; and (4) compare the influences of MFDA use between frequent and infrequent users.

## Methods

### Research Design

This cross-sectional study adopted an explanatory sequential mixed methods research design to provide a comprehensive understanding of the research topic, which cannot be obtained through the conduct of a single quantitative or qualitative study [16-18].

The first 2 research objectives can be aptly addressed using a quantitative design, whereas the last 2 research objectives can be adequately answered through a qualitative design. In the *Discussion* section, the results from both approaches will be integrated to comprehensively respond to the research questions and establish a complete understanding of the research topic.

In the quantitative component, participants completed a questionnaire, and in the qualitative component, a subset of participants took part in semistructured in-depth interviews. To guarantee the quality and appropriate interpretation of the data, the researchers followed the CHERRIES (Checklist for Reporting Results of Internet E-Surveys) guidelines [19].

### Study Population and Sampling Method

Participants from National University of Singapore (NUS) were recruited to complete the web-based questionnaire via convenience sampling. The eligibility criterion was students aged 18 to 35 years. The research team sent an email to each teaching department in NUS. The departments that agreed to participate forwarded this email to their students. This email contained a general link to a REDCap (Research Electronic Data Capture; Vanderbilt University) web page [20,21]. Anyone with the link could access the questionnaire.

The selection criteria for the qualitative participants were the same as the eligibility criteria for the quantitative design. The study intentionally recruited individuals who used MFDAs more frequently compared with those who used them less often to gather insights and feedback on MFDA use patterns. In addition, these participants were selected via purposeful stratified sampling, reflecting the age, sex, and racial profile of young adults in Singapore [22]. The quota sampling frame is presented in Multimedia Appendix 1.

### Sample Size

Using a sample size calculator [23] and based on an expected prevalence rate of 25%, 95% CI, precision of 5%, and a population size of 38,607 NUS students [24], the estimated sample size for this study was 286. However, to minimize the likelihood of type-2 errors, 480 participants were expected to be recruited. Notably, a similar study examining the prevalence of healthy eating among university students also used this sample size to achieve statistically relevant results [4]. The interviews were conducted until data saturation was reached.

### Development of the Questionnaire

#### Overview

One of the researchers (XCT) created a sample questionnaire based on the literature [4,25-27], which was then sent to public health nutrition experts to check for construct and content validity. Once their suggestions were incorporated, XCT piloted the web-based questionnaire with a diverse group of students (n=5) to check if the questions were understood by them and see if it could be completed within a reasonable time. Their responses to the questionnaire were not included as data. The 21-item questionnaire was then finalized by the research team (Multimedia Appendix 2). Saw Swee Hock School of Public Health (SSHSPH), the faculty where XCT was a student, used the REDCap platform to create and administer the questionnaire. The server responsible for hosting the platform was also maintained by SSHSPH [20,21]. REDCap provides a secure method for creating and managing surveys and databases through a web-based platform [20,21].

#### Exposure and Intermediate Outcome Variable

Participants were asked questions about the frequency of MFDA use. A 10-point Likert scale ranging from “Never or rarely” to “6+ a day” was adapted from a diet screener [26]. It asked questions about the frequency of eating food items commonly consumed by the Asian population. High-frequency use was defined as at least once a week, whereas low-frequency use was defined as less than once a week. A similar study also dichotomized frequency of use in this way and found that there was 85% agreement for test-retest reliability [28].

Questions about other patterns included changes in MFDA use compared with before the COVID-19 pandemic, the period in which participants tended to purchase more often, and the types of cuisines commonly ordered.

#### Outcome Variables

##### Fruit and Vegetable Consumption

Fruit and vegetable consumption was assessed using the following questions: (1) How many servings of fresh fruits do you eat on a typical day? and (2) How many servings of vegetables do you eat on a typical day? The choices were presented as an 8-point Likert scale ranging from “0” to “>6.” The Likert scale was adapted from the Singapore National Health Survey 2010 [27]. These questions have also been used in previous studies involving Singapore university students [4]. The combined fruit and vegetable consumption was calculated by adding the servings of fruits and vegetables consumed per day. Singapore’s national dietary guidelines recommend 2 servings of fruits and 2 servings of vegetables per day [3]. Thus, participants consuming ≥4 servings of fruits and vegetables per day were categorized as meeting the guidelines.

##### SSB Consumption

SSBs in this study were defined as any beverage that contained added sugar, including low-sugar or low-caloric drinks. SSB consumption was assessed using the following question: “How many servings of sugar-sweetened beverages do you consume on a typical day?” The choices were presented as an 8-point Likert scale ranging from “0” to “>6” adapted from the study by Robertson et al [25]. A similar question was used in a healthy diet study among adults [27]. Singapore’s dietary guidelines encourage choosing water to drink [3]; therefore, we considered participants who consumed 0 servings of SSBs per day to meet this guideline.

##### BMI Measurement

The self-reported height and weight of the participants were recorded. BMI was calculated as 
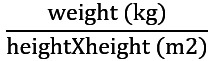
. A healthy BMI for Asian populations is defined as <23.0 kg/m^2^ [7].

#### Covariates

##### Leisure-Time Physical Activity Level

A question about leisure-time physical activity level was adapted from the Singapore National Health Survey 2010 (“In the past 3 months, did you participate in any sports, exercise or walking during your leisure time?”) [27].

##### Demographics

Participants’ demographics (including age, sex, race, marital status, number of children, working status, student status [undergraduate or graduate and nongraduating students], and study status [full-time or part-time study]) were collected.

### Development of Interview Guide

The researchers formulated the initial questions of the interview guide to explore the research questions in an open-ended, inductive way (Textbox 1; see Multimedia Appendix 3 for the full guide). The initial open questions and probes allowed the interviewees to elaborate on their responses. As the interview progressed, the questions were more deductive and used the questionnaire as a framework. Drawing from the quantitative survey results, the questions aimed to shed light on the patterns of MFDA use. For instance, the survey revealed that approximately 60% of the participants used MFDAs infrequently. On the basis of the interviewees’ frequency of use, the researchers asked for their opinions on the survey results. Multimedia Appendix 3 contains similar questions following this approach. Participants were encouraged to elaborate further if their responses were beyond the scope of the guide.

The guide was vetted by public health nutritionists and reviewed by an academic whose teaching and research involve qualitative methods. XCT pilot-tested the guide on a university student, and further changes were made before it was sent for ethics approval.

**Summary of the interview guide.**

**Factors of mobile food delivery app (MFDA) use**

- What are the factors that drive frequent or infrequent use of MFDAs?
  Probe: in your opinion, what are the factors that drive your personal use of MFDAs? Why do you think so?
- How did your use patterns change during the COVID-19 pandemic?
  Probe: how has your use of MFDAs changed from the pre–COVID-19 period to the current situation?
- What attracts you to use certain MFDAs?
  Probe: what are the factors that entice users to use certain food apps?
- Why are there high use patterns observed for lunch and dinner?
  Probe: as compared with eating out, what are the enticing factors for one to buy meals via MFDAs?

**Healthfulness of food ordered via MFDAs**

- Why is food ordered via MFDAs mostly unhealthy?
  Probe: do you feel that young adults purchase healthier or less healthy meals through the apps? What are the possible reasons?
- Why do you think that the food ordered cannot meet the dietary requirements of fruits and vegetables?
  Probe: do the foods purchased from the apps contain adequate fruits and vegetables? Why is this so?
- How do users decide to purchase specialty drinks via MFDAs?
  Probe: what might entice users to purchase sugar-sweetened beverages through the apps?

**Physical activity level when using MFDAs frequently**

- How has the frequent use of MFDAs affected your level of physical activity?
  Probe: how has the physical activity level of MFDA users changed because of the convenience of food delivery?

**Future or current use of MFDAs**

- How will your use patterns change once you start working?
- How will your use patterns change once you are married and have children?

### Ethics Approval and Informed Consent

This study was approved by NUS SSHSPH Department Ethics Review Committee (SSHSPH-056). All participants were required to provide written informed consent to take part. The data collected were coded to keep the identities of the participants confidential.

### Data Collection Methods

Responses to the questionnaires were collected from January 14, 2021, to April 30, 2021, whereas qualitative interviews were conducted from March 18, 2021, to May 21, 2021, during a less restrictive phase of Singapore’s COVID-19 measures.

Before the start of the questionnaire, potential participants were informed that taking part in this project was completely voluntary and that their responses would contribute to the understanding of a new area relevant to promoting healthy eating among young adults. Potential participants were also given a guarantee of anonymity and were informed that only XCT and his research supervisors, FM-R and NAP, would have access to the raw data. The expected time to complete the questionnaire was 15 minutes. The end of the survey asked for their contact details if they were interested in participating in the qualitative interviews in the future. Once the participants had submitted their responses, they were unable to modify their answers. There were no cookies saved on the electronic devices of the participants, and the REDCap servers did not store any IP addresses. Participants were not given any reimbursements for completion of the questionnaire.

XCT conducted the face-to-face interviews via Zoom (Zoom Video Communications) [29]. All interviewees were informed that their responses would be confidential and audio recorded before the interviews started. Field notes were also recorded to ensure the credibility of the data. Participants received a S $20 (US $15) food delivery e-voucher each after the interviews. Each interview lasted approximately 40 to 60 minutes. Data continued to be collected until data saturation, when no new themes emerged, was reached on the 19th interview.

### Analysis

All quantitative data were analyzed using Stata (version 17; StataCorp) [30]. Descriptive analysis was first conducted. Chi-square and Poisson regression analyses were used to investigate any associations between the categorical variables; *P*<.05 was considered statistically significant. A backward stepwise method was used in the regression analysis to select covariates to account for any possible confounding factors to obtain the adjusted prevalence ratio (aPR) and CI [4].

All the interviews were transcribed verbatim. Qualitative data were analyzed using NVivo (QSR International) for Windows [31]. The thematic analysis was guided by the 6-phase approach by Braun and Clarke [32]. XCT first familiarized himself with the data (phase 1) by reading the transcripts and listening to the audio recordings. XCT independently coded and analyzed the transcripts (phase 2) using the interview guide as a framework for analysis. Semantic themes were first derived from the interview guide (phase 3). After the analysis of the first 2 transcripts, the themes were further reviewed and modified to fit with the codes (phase 4). XCT also took a reflexive approach by writing a research diary every 5 transcripts to reflect on how his experiences of using MFDAs and as a young adult could affect the analysis and data collection [33]. To progress to latent themes, XCT referred to parallel literature on takeout consumption patterns and cooking patterns among young adults to obtain extensive interpretation (phase 5) [34,35]. XCT also met with NAP via Zoom at every phase to ensure that the interpretations of the data truly reflected what the participants had said and reach a consensus on the themes. NAP also provided input on and reviewed the overall findings presented on 2 occasions after phase 5. After the fifth phase, as recommended by NAP, the fourth research objective was added. To answer this question, XCT used the “Framework Matrix” function in NVivo; frequent and infrequent users were grouped into rows, and the themes and subthemes were grouped into columns. This function facilitated a comparison of the feedback of frequent versus infrequent MFDA users and facilitated a synthesis of the data in table format. The themes were thought through and analyzed to form an overall narrative of MFDA use patterns (phase 6). These 6 phases were always in iteration; XCT moved back and forth between phases. The final synthesis used a narrative-weaving approach to integrate the quantitative and qualitative findings [36]. Fetters et al [36] suggested that presenting quantitative and qualitative findings together based on their corresponding themes is a sound approach. The “fit” of data integration refers to the coherence between these 2 types of data, which can lead to 1 of 3 possible outcomes. The first outcome, confirmation, is when the findings from both data sources support each other, thus providing greater credibility to the results. The second outcome, expansion, occurs when the 2 data sources offer divergent insights into the phenomenon of interest, providing a more comprehensive understanding of it. The third outcome, discordance, occurs when there is inconsistency or incongruity between the qualitative and quantitative findings. In such cases, potential sources of bias and methodological assumptions and procedures should be examined. To further illustrate the relationships between the data sets, meta-inferences are presented.

## Results

### Demographic Profile of the Participants

**Figure 1:**
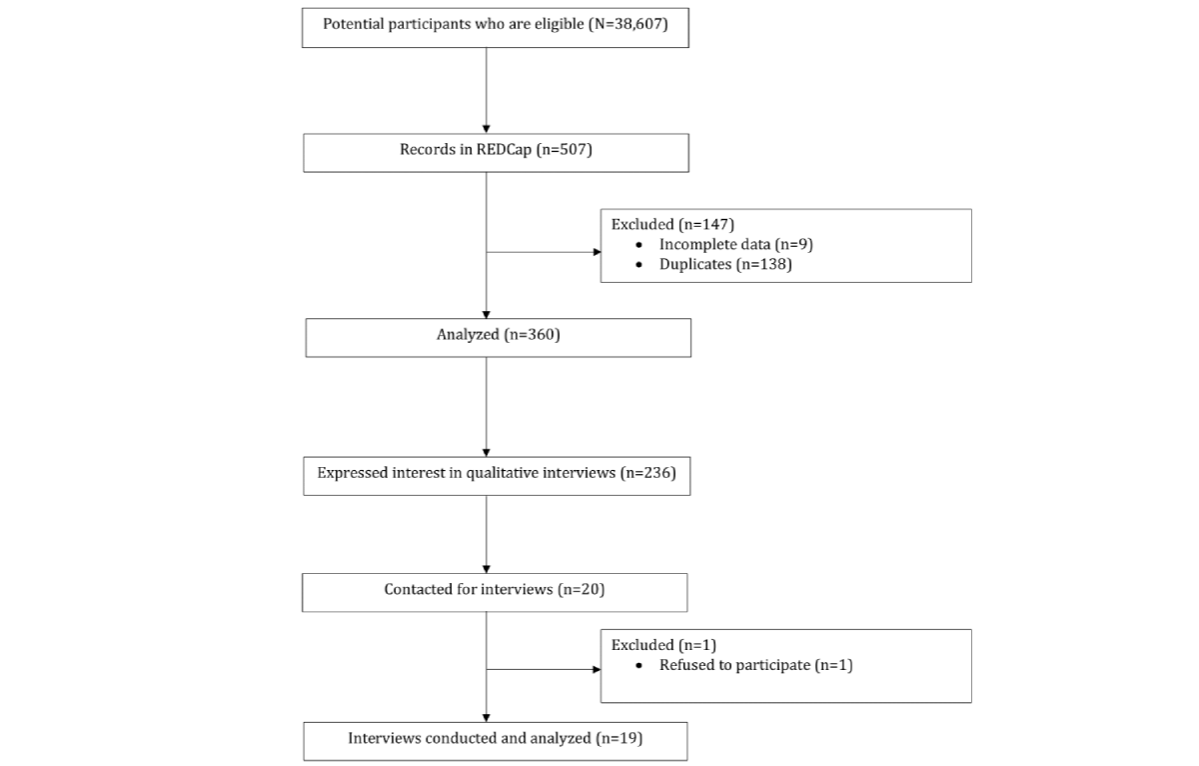
Study flow diagram of how the participants were selected. REDCap: Research Electronic Data Capture.

Of the 507 entries recorded in REDCap, 360 (71%) responses were retrieved for analysis after removal of duplicates (n=138, 27.2%) and incomplete data (n=9, 1.8%; see Figure 1 for the flowchart). It is worth noting that the high number of duplicates can be attributed to the participants’ enthusiasm to take part in the interview, which could be attributed to the S $20 (US $15) incentive, which was more substantial than the incentive offered in previous studies conducted at NUS. The response rate for the survey was 0.93% (360/38,607).

Table 1 presents the participants’ characteristics. The median age of the survey participants was 23 (range 21-25) years. A total of 69.2% (249/360) of the participants identified as female. Most survey participants (308/360, 85.6%) were Chinese. In comparison with the survey participants, the interviewees had a median age of 25 (range 23.0-27.5) years. There were 53% (10/19) of female participants in the sample, and most (13/19, 68%) were Chinese (see Multimedia Appendix 1 for the stratification of the interviewees).

**Table 1:** Participants’ demographic profile.

Characteristic			Survey participants (n=360)		Interview participants (n=19)
**Age (years), median (IQR)**			23 (21-25)		25 (23.0-27.5)
	18-23, n (%)	233 (64.7)		6 (31.6)	
	24-29, n (%)	94 (26.1)		10 (52.6)	
	30-35, n (%)	33 (9.2)		3 (15.8)	
Female participants, n (%)			249 (69.2)		10 (52.6)
Chinese participants, n (%)			308 (85.6)		13 (68.4)
Not working, n (%)			276 (76.7)		11 (57.9)
Single, n (%)			337 (93.6)		18 (94.7)
No children, n (%)			352 (97.8)		18 (94.7)
Undergraduates, n (%)			248 (68.9)		7 (36.8)
Full-time study, n (%)			314 (87.2)		15 (78.9)

### Quantitative Findings

#### Objective 1: Examine the Use Patterns of MFDAs Among Young Adults During the COVID-19 Pandemic

Table 2 displays MFDA use patterns. MFDA use frequency is grouped into 3 categories: 58.3% (210/360) of the participants mentioned that they used MFDAs less than once a week, whereas 19.4% (70/360) used MFDAs once a week and 22.2% (80/360) used MFDAs at least twice per week. Most participants (250/307, 81.4%) also used MFDAs more often compared with before the pandemic. Lunch (224/584, 38.4%) was the most commonly bought meal. Asian-based cuisines (249/843, 29.5%) and fast food (225/843, 26.7%) were the most commonly bought meal types.

**Table 2:** Mobile food delivery app use patterns (n=360).

			Participants, n (%)
**How often do you use mobile food delivery apps to purchase food? (n=360)**			
	**Less than once a week**		210 (58.3)
		Never or rarely	53 (14.7)
		Once a month	63 (17.5)
		2-3 times a month	94 (26.1)
	Once a week		70 (19.4)
	**At least twice per week**		80 (22.2)
		2-3 times a week	48 (13.3)
		4-6 times a week	20 (5.6)
		Once a day	6 (1.7)
		2-3 times a day	4 (1.1)
		4-5 times a day	2 (0.6)
**Do you notice yourself using mobile food delivery apps more often than before the COVID-19 pandemic started? (n=307)^a^**			
	No		57 (18.6)
	Yes		250 (81.4)
**During which period or meal do you usually purchase food via mobile food delivery apps? (You may choose more than 1 option; n=584)**			
	Breakfast		26 (4.5)
	Lunch		224 (38.4)
	Tea break		44 (7.5)
	Dinner		220 (37.7)
	Night snacks		70 (12)
**What type of cuisine do you usually order from mobile food delivery apps? (You may choose more than 1 option; n=843)**			
	Beverages		109 (12.9)
	Salads		15 (1.8)
	Desserts		55 (6.5)
	Asian-based		249 (29.5)
	Fast food		225 (26.7)
	Western-based		182 (21.6)
	Convenience food (eg, microwavable food and instant noodles)		8 (0.9)

^a^Missing values because of branching of question.

#### Objective 2: Investigate the Association Between MFDA Use and Sociodemographic Factors, Dietary Factors, and BMI

##### Association of Frequency of MFDA Use With Sociodemographic Variables

A higher percentage of female participants used MFDAs less frequently compared with their male counterparts (150/249, 60.2% vs 60/111, 54.1%), whereas a higher percentage of graduates and nongraduating students were more likely to use MFDAs frequently compared with undergraduates (once a week: 28/112, 25% vs 42/248, 16.9%; at least 2 times per week: 28/112, 25% vs 52/248, 21%). However, none of these differences were statistically significant (Table 3).

**Table 3:** Sociodemographic variables associated with frequency of mobile food delivery app (MFDA) use (n=360).

		Frequency of MFDA use, n (%)				Chi-square (*df*)			*P* value	
		Less than once a week	Once a week	At least 2 times per week						
**Age (years)**							4.0 (4)			.41
	18-23	142 (60.9)	41 (17.6)	50 (21.5)						
	24-29	47 (50)	23 (24.5)	24 (25.5)						
	30-35	21 (63.6)	6 (18.2)	6 (18.2)						
**Sex**							3.0 (2)			.22
	Nonfemale^a^	60 (54.1)	20 (18)	31 (27.9)						
	Female	150 (60.2)	50 (20.1)	49 (19.7)						
**Race**							5.0 (2)			.08
	Non-Chinese	29 (55.8)	6 (11.5)	17 (32.7)						
	Chinese	181 (58.8)	64 (20.8)	63 (20.5)						
**Marital status**							0.8 (2)			.69^b^
	Married	13 (56.5)	6 (26.1)	4 (17.4)						
	Single	197 (58.5)	64 (19)	76 (22.6)						
**Number of children**							2.0 (2)			.30^b^
	≥1	3 (37.5)	3 (37.5)	2 (25)						
	0	207 (58.8)	67 (19)	78 (22.2)						
**Working status**							1.6 (2)			.45
	Yes	48 (57.1)	20 (23.8)	16 (19.1)						
	No	162 (58.7)	50 (18.1)	64 (23.2)						
**Student status**							5.1 (2)			.08
	Graduate and nongraduating	56 (50)	28 (25)	28 (25)						
	Undergraduate	154 (62.1)	42 (16.9)	52 (21)						
**Study load**							4.2 (2)			.12
	Part-time	24 (52.2)	14 (30.4)	8 (17.4)						
	Full time	186 (59.2)	56 (17.8)	72 (22.9)						

^a^Includes 2 participants who identified as nonbinary (queer).

^b^Fisher exact test was used.

##### Association Between Frequency of MFDA Use and Fruit and Vegetable Consumption, SSB Consumption, and BMI

Multimedia Appendix 4 presents the univariate analysis of the associations between frequency of MFDA use and fruit and vegetable consumption, SSB consumption, and BMI. Associations between frequency of MFDA use and fruit and vegetable consumption, SSB consumption, and BMI were also not statistically significant in the adjusted model of regression analysis (Table 4); however, for selected outcomes such as vegetable and SSB consumption, a trend toward an association was observed.

Those who used MFDAs at least twice per week reported a 12% lower frequency of consuming 2 servings of vegetables per day compared with those who used MFDAs less than once a week (aPR 0.88, 95% CI 0.63-1.24).

Using “less than once-a-week” as a reference, those who used MFDAs at least twice per week (aPR 0.89, 95% CI 0.57-1.38) and those who used MFDAs once a week (aPR 0.62, 95% CI 0.37-1.38) also reported lower prevalence of not consuming any servings of SSBs per day (11% and 38%, respectively; Table 4).

Chi-square trend tests revealed no dose-response relationships among frequency of MFDA use, fruit and vegetable intake, SSB intake, and BMI.

**Table 4:** Adjusted prevalence ratio (aPR) of frequency of mobile food delivery app (MFDA) use association with diet and BMI (n=360).

Frequency of MFDA use			aPR (95% CI)		*P* value for trend
**2 servings of fruit intake per day^a^**					.76
	Less than once a week	Reference			
	Once a week	0.95 (0.55-1.65)			
	At least 2 times per week	0.93 (0.55-1.56)			
**2 servings of vegetable intake per day^a^**					.24
	Less than once a week	Reference			
	Once a week	0.99 (0.69-1.40)			
	At least 2 times per week	0.88 (0.63-1.24)			
**4 servings of combined fruit and vegetable intake per day^a^**					.85
	Less than once a week	Reference			
	Once a week	0.94 (0.67-1.32)			
	At least 2 times per week	1.05 (0.76-1.43)			
**0 servings of sugar-sweetened beverage intake per day^b^**					.22
	Less than once a week	Reference			
	Once a week	0.62 (0.37-1.04)			
	At least 2 times per week	0.89 (0.57-1.38)			
**BMI of <23 kg/m^2^^c^**					.09
	Less than once a week	Reference			
	Once a week	1.00 (0.73-1.37)			
	At least 2 times per week	0.91 (0.66-1.25)			

^a^Adjusted for the following variables: age, sex, race, marital status, number of children, current working status, student status, study load, participation in physical activity, BMI, and sugar-sweetened beverage intake.

^b^Adjusted for the following variables: age, sex, race, marital status, number of children, current working status, student status, study load, participation in physical activity, BMI, and combined fruit and vegetable intake.

^c^Adjusted for the following variables: age, sex, race, marital status, number of children, current working status, student status, study load, participation in physical activity, sugar-sweetened beverage intake, and combined fruit and vegetable intake.

### Qualitative Findings

#### Objective 3: Identify the Underlying Reasons for the Observed Use Patterns of MFDAs Among Users

##### Overview

To complement the survey data, 19 participants were purposefully selected to ask them about their perceptions and experiences of using MFDAs. A total of 84% (16/19) of the interviewees were frequent users.

**Figure 2:**
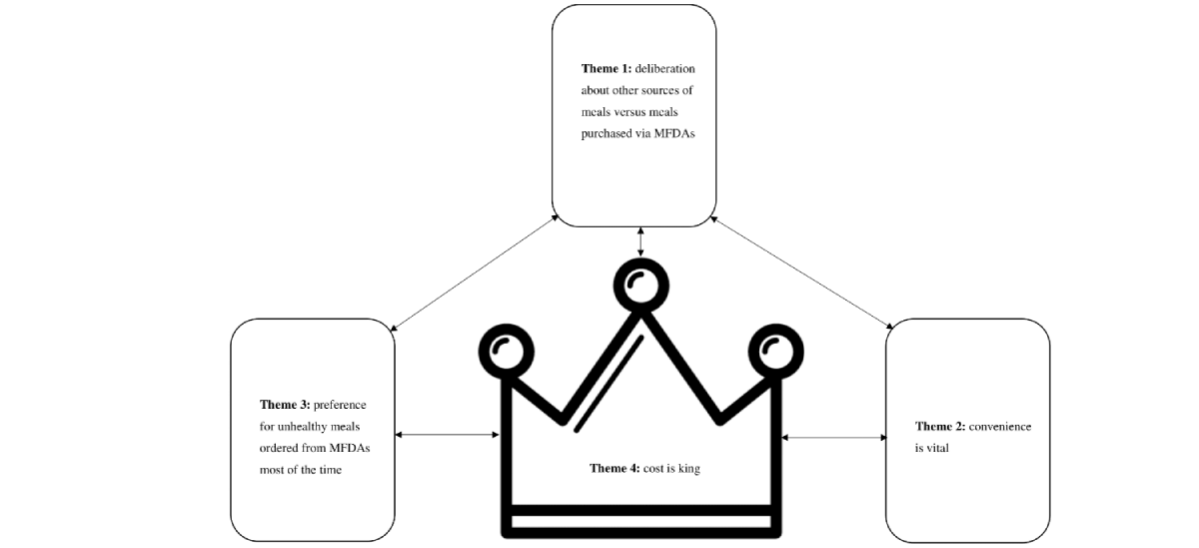
Conceptual framework that describes the factors that users considered before using mobile food delivery apps (MFDAs).

There were 4 main themes identified from the analyses (see Multimedia Appendix 5 for other quotes). Individuals will consider several factors in their decision-making process (themes 1 to 4) before making the ultimate decision to use MFDAs. Considering the first theme, “deliberations about other sources of meals versus meals purchased via MFDAs,” individuals would contemplate if they were willing to obtain meals by cooking or buying meals outside their homes; if not, they would order in. In the discussion related to the second theme, “convenience is vital,” individuals would weigh conveniences such as time and staying at home against the costs of purchasing food via MFDAs. In the third theme, “preference for unhealthy food ordered from MFDAs most of the time,” individuals relayed wanting to have a sense of satisfaction by consuming unhealthy food and made statements reflecting that the national dietary recommendations were not an important factor in decisions about food. The ultimate determinant in the decision-making process was the cost of food delivery, as indicated by the fourth theme, “cost is king,” which could take precedence over any of the previously mentioned considerations. The “cost” in this finding is represented in dollars and cents. Both vendor selection and discount hunting were important factors in the purchasing decisions of young adult MFDA users. The identified themes were characterized by a recursive decision-making process among users as they weighed each theme in a back-and-forth manner before finalizing their food purchases via MFDAs. Figure 2 presents a conceptual framework based on these themes, where each box denotes a particular theme that users consider before making a decision and the 2-way arrows depict the iterative thought process that occurs. It is important to note that users did not consider the themes in sequence; for example, they did not consider theme 1 first, followed by theme 2, and so on, but rather, they considered all factors simultaneously. Notably, the cost factor is represented by a crown symbol instead of a box as it holds the greatest importance in purchasing decisions.

##### Theme 1: Deliberations About Other Sources of Meals Versus Meals Purchased via MFDAs

Before using MFDAs, individuals described considering how they could obtain meals from sources such as inside or outside their homes.

###### Subtheme 1.1: Homemade Meals

For most interviewees, cooking at home was the most discussed concept. If there were homemade meals, they would not use MFDAs. The action of cooking was mostly carried out by themselves or their parents:

...[I don’t use MFDAs often because my dad]...cook for me...I will just...cook something like vegetables and stuff.

MFDA121; infrequent

Similarly, if there was no one to cook meals, MFDAs would be used:

...we don’t want to...cook and don’t want to trouble my parents to cook, so we’ll just like order [via MFDAs].

MFDA121; infrequent

###### Subtheme 1.2: Outside Meals

Meals could also be obtained from outside the individuals’ homes. They might dine in, purchase takeout, or even choose pick-up options from MFDAs.

As COVID-19 restrictions regarding attending places of work or study and dining out eased, there were fewer opportunities to use MFDAs:

...because if you’re working outside, you are already outside, so you might as well just go and buy some food from nearby...

MFDA418; frequent

##### Theme 2: Convenience Is Vital

MFDA users appreciated the convenience of ordering food. They may need time to finish their work or simply refuse to step out of their homes. Whichever convenience they require justified the use of MFDAs.

###### Subtheme 2.1: Time is of the Essence

Being students, interviewees required time to complete their assignments and revise for exams:

I want to spend more time in study. So I want to save time then I go order.

MFDA425; frequent

Similarly, working adults might face time pressure to finish their tasks:

...if it is quite urgent that I...finish the task, I will order online.

MFDA147; frequent

Most interviewees were studying and working during the daytime; they would order lunch so that they had more time to complete their tasks of the day. For dinner, they would have more time to prepare their food:

...people are busy during lunch times. They’re either doing work or doing assignments...have some meeting...or rushing from one place to another. At dinner...I have time to...cook...

MFDA471; frequent

###### Subtheme 2.2: Staying at Home

Staying at home is a convenience. Most individuals did WFH and had web-based lessons for extended periods close to or during the time they were interviewed. These interviewees expressed that they simply did not see the need to step out of their homes for that day as they were already at home:

I’m at home, that’s why I don’t want go out.

MFDA425; frequent

As individuals were not stepping out of their homes to procure their meals, there was less probability that users would head out to exercise:

...if...I go and purchase [MFDAs]...I usually don’t do any manual work. Like I don’t do any exercise...So if I just sit here and order delivery...I...feel lazy.

MFDA29; frequent

##### Theme 3: Preference for Unhealthy Meals Ordered From MFDAs Most of the Time

Although young adults understood the importance of the national dietary requirements, they often purchased unhealthy meals from MFDAs:

When they [my friends] order Poke [a salad bowl] [via MFDAs]...I didn’t order Poke with them, and I really appreciated the healthy thing...but for myself, I am highly resistant...I’d like to have something good.

MFDA346; frequent

###### Subtheme 3.1: Consuming an Unhealthy Diet Is Personally Satisfying

The decision to purchase unhealthy meals related to a sense of self-satisfaction. For many individuals, satisfaction meant relieving hunger:

...[if I am] getting the pizza, I'm getting a garlic bread stick and...a Coke. And if I’m really hungry then I will give myself the concession to go unhealthy and order them...because I know that a salad in the same price [as the pizza combo] will necessitate that I order something else again and again...

MFDA176; frequent

For others, satisfaction meant feeling relaxed after stressful events such as work, school assignments, and exams. They would comfort or reward themselves by buying unhealthy options via MFDAs:

I think when I’m stressed...I just want to please myself, I want the instant gratification from the nice food.

MFDA234; frequent

###### Subtheme 3.2: National Dietary Recommendations Are Not an Important Factor in Decisions Regarding Food

Interviewees argued that it was unimportant to adhere to the national dietary recommendation of fruit and vegetable consumption when using MFDAs; rather, it was an individual’s preference to be health conscious:

...I don’t think...when I order, am I really thinking that I need to fulfil the national dietary requirements...I don’t think that’s how I’m thinking while I’m ordering. I’m thinking like...where is good food?

MFDA176; frequent

...if you want to eat healthy it’s kind of like an intentional thing. Like you already know that you want to be healthy and you plan for it.

MFDA418; frequent

##### Theme 4: Cost Is King

###### Overview

Cost refers to the final cost of the food ordered via MFDAs after the deduction of any prevailing discounts. This theme played the most crucial role in deciding a user’s purchase. If the total cost was low enough, it might override other deliberations described under the preceding themes and have a negative impact on health:

...the delivery fee is much...cheaper...back in China...I used the food delivery every day, maybe for every meal...I was gaining weight...I don’t do the physical exercise...I think I become a couch potato...But in Singapore...the food delivery won’t get so much influence on my health.

MFDA481; frequent

This theme was consistent with the observations of infrequent users, who indicated that the main obstacle to MFDA use was the exorbitant price:

I think...the delivery charges and also the cost on the app itself, for certain food items are like higher. So I think that kind of outweighs...the convenience of getting food from the app.

MFDA341; infrequent

If the cost of ordering from MFDAs was excessively high, frequent users might even decrease their reliance on MFDAs to the point where they became infrequent users:

...back in Indonesia, I rarely cook...I tend to order from delivery app...I think cost...influenced me back then...when the delivery app is...popular in Indonesia, the cost of the food [via MFDAs] is worth a lot cheaper...they [MFDA companies] just want to introduce...so many discounts. But in Singapore, I rarely use the app.

MFDA377; infrequent

###### Subtheme 4.1: Cost Considerations When Choosing Vendors

Users would contemplate the total cost of food when choosing food vendors as the food ordered via MFDAs was often expensive to them. Considerations included whether the price of food and the delivery fee were within their budget. If not, they would choose other vendors:

...the first idea is always look at the price of the food. If that’s high...[I’ll]...choose something else. If...the criteria of food price [is fulfilled], the next idea would be...minimum order...let’s say...if I pass,...I see the delivery charge...If it’s reasonably priced...why not?

MFDA471; frequent

###### Subtheme 4.2: Strategies to Reduce Cost

There were various ways to reduce the costs of food delivery. A commonly used strategy was to search for discounts that offered cheaper food or delivery fees. These discounts may appear in some food vendors, in different MFDA platforms, or in the form of promotional codes. Regardless, these discounts would be attractive enough to determine food purchases:

I look for discounts first before [I] search for the food...see whatever is...cheap delivery.

MFDA418; frequent

#### Objective 4: Compare the Influences of MFDA Use Between Frequent and Infrequent Users

The framework matrix comparing frequent and infrequent MFDA users revealed that most frequent users could not cook meals for themselves:

I...know...people of our generation don’t really cook.

MFDA38; frequent

Instead, frequent users tended to depend on their parents to cook:

...I don’t cook, because...my mother cooks.

MFDA409; frequent

In contrast, infrequent users or their parents tended to cook food often. Nonetheless, it is important to recognize that infrequent users might still opt to purchase particular food items from MFDAs if they are incapable of preparing these meals on their own:

...I cannot cook that specific food...for example Mala (麻辣) [commonly used Chinese word to describe for “numbing and spicy” food]...and Bee hoon [rice vermicelli]...and Nasi Lemak [a Malay cuisine] then I will order.

MFDA377; infrequent

Although not explicitly mentioned, frequent users expressed that they were negatively affected by the COVID-19 restrictions, which resulted in the consumption of extra food or an unhealthy diet:

...[When I was] in quarantine, SHN [Stay-Home-Notice]...[I] start getting hungry at weird times. [I had] some weird cravings...[I] start getting hungry at midnight, even if [I] had lunch and dinner that [the hotel] provided.

MFDA471; frequent

...during Circuit Breaker, I’m not as healthy. I ordered bubble tea, I ordered ice cream, I ordered fast-food, I ordered unhealthy food...[because] I feel very sad...[that I] cannot go out...Then [I] eat to be happy lor (a Singaporean colloquial term used to express resignation).

MFDA409; frequent

However, infrequent users were not particularly affected by the restrictions.

## Discussion

### Principal Findings

This study aimed to investigate the use patterns of MFDAs among young adults during the COVID-19 pandemic; examine the association between MFDA use and sociodemographic factors, dietary factors, and BMI; identify the underlying reasons for the observed use patterns of MFDAs among users; and compare the influences of MFDA use between frequent and infrequent users. The quantitative findings showed that 41.7% (150/360) of the participants used MFDAs frequently. Other established use patterns included MFDAs being used more often compared with before the pandemic, lunch being the most commonly bought meal, and Asian-based cuisines and fast food being common food choices. Sex and student status were found to have empirical relevance to the frequency of MFDA use, but this association was found to be not statistically significant. Regression analysis revealed that frequent users had a lower prevalence of consuming 2 servings of vegetables per day and a higher prevalence of drinking SSBs compared with infrequent users, although the differences between frequent and infrequent users were not statistically significant. In total, 4 overarching themes emerged from the qualitative analysis: deliberations about other sources of meals versus ordering in, convenience is vital, preference for unhealthy food ordered from MFDAs most of the time, and cost is king. The framework matrix revealed that the lack of culinary skills and COVID-19 restrictions influenced frequent use.

Taking both the quantitative and qualitative findings into consideration, 4 meta-inferences emerged and will be discussed alongside the existing literature and practical implications. The researchers adopted a narrative-weaving approach that integrates findings from both designs on a concept-by-concept approach [36]. Meta-inferences derived from the narrative-weaving approach are shown in Table 5, indicating the seamless integration of the various data sets with no detectable inconsistency or incongruity (discordance) in the findings.

**Table 5:** Meta-inferences derived from the findings.

Quantitative findings	Qualitative findings derived from thematic analysis	Qualitative findings derived from framework matrix	“Fit” of data integration (confirmation, expansion, or discordance)	Meta-inferences
Most of the participants used MFDAs^a^ more often compared with before the COVID-19 pandemic.	Convenience is vital	COVID-19 restrictions	Expansion	Staying at home is a double-edged sword as it is convenient but can lead to unhealthy behaviors.
Female participants used MFDAs less frequently than male participants.	Deliberations about other sources of meals versus meals purchased via MFDAs	Lack of culinary skills	Expansion	Cooking is valued by female participants.
Fast food was one of the most commonly bought meal types. Frequent users were less likely to consume 2 servings of vegetables per day and more likely to drink SSBs^b^. Graduates and nongraduating students were more likely to use MFDAs frequently compared with undergraduates.	Preference for unhealthy foods ordered from MFDAs most of the time	—^c^	Confirmation	Young adults’ general indifference toward healthfulness of diet
41.7% of survey participants used MFDAs frequently. Lunch was the most commonly bought meal.	Cost is king	—^c^	Confirmation	Cost of food delivery is trivial if time is scarce.

^a^MFDA: mobile food delivery app.

^b^SSB: sugar-sweetened beverage.

^c^No suitable findings for data integration.

### Staying at Home Is a Double-edged Sword as It Is Convenient but Can Lead to Unhealthy Behaviors

The main purpose of the COVID-19 restrictions was to prevent the spread of the virus. However, they brought about the secondary benefit of staying at home. It was found that self-interest plays a role in compliance with restrictions [37]. The quantitative findings revealed that most participants (250/307, 81.4%) used MFDAs more frequently during the COVID-19 pandemic compared with before the pandemic, which was corroborated by the qualitative interviews.

In the qualitative component, interviewees mentioned that, while doing WFH and learning on the web, they tended to use MFDAs often as they could enjoy the convenience of staying at home, which corroborated the survey findings that individuals used MFDAs more frequently compared with before the pandemic. The qualitative findings also revealed that frequent MFDA users did not feel like engaging in any physical activity outside their homes as COVID-19 restrictions bred sedentary behaviors among young adults [38]. A hybrid mode of working remotely and in the office is likely to be part of the *new normal* for work [39]; hence, this is an important consideration in the population’s physical activity levels and dietary patterns. Future interventions can focus on young adults who are tasked with staying at home. Suggestions include moving around for 2 minutes for every 30 minutes of sitting to interrupt sedentary time [40]. Smartphones have been shown to be great platforms for promoting physical activity, such as sending reminders to users and sharing workout achievements with others. Interventions can use existing resources such as Facebook, YouTube, and Zoom “live” sessions in compliance with social distancing guidelines [41].

COVID-19 restrictions had unintended impacts on individuals. Some of the interviewees experienced cabin fever because of staying at home for long durations, which, according to one study, could alter eating patterns [38]. Cabin fever explains some interviewees’ frequent MFDA use during home isolation. Interventions could emphasize mindfulness meditation of being aware of the surroundings, such as keeping a calendar in sight, and seeking professional help if stress, anxiety, or loneliness are experienced [42].

### Cooking Is Valued by Female Participants

The qualitative interviews revealed that frequent MFDA users did not cook often. It was suggested in a US study with millennials that they did not have sufficient culinary skills; thus, they preferred to dine in or buy takeout [34]. It is possible that these young adults did not develop cooking behaviors from their parents when they were living with them as previous research has found this to be the case [35]. Hence, they depended on their parents to cook their meals. Moreover, the quantitative component also highlighted that a higher percentage of female participants were less likely to use MFDAs compared with their male counterparts. Possible explanations for this could include that studies have suggested that female individuals generally have better cooking skills than male individuals [43,44], which might reduce their need to rely on MFDAs for obtaining food. In addition, female individuals may be more likely to cook their meals frequently to prioritize healthier eating habits. Frequent cooking is significantly associated with better diet quality [45], which suggests that female individuals deliberately choose to eat healthily according to the qualitative findings. This suggestion was also supported by a scoping review, which found sex differences in healthy eating, including female participants being more aware of their body image; hence, they are more likely to eat healthily compared with male individuals [46].

Interventions should focus on cultivating cooking habits, especially among young male adults. Before habits are formed, young adults should have confidence in their culinary skills [47]. As such, cooking skill programs can focus on teaching young adults, especially male individuals, basic food preparation skills to boost confidence [48].

### Young Adults’ General Indifference Toward Healthfulness of Diet

Young adults generally have a nonchalant attitude toward healthy eating [46], believing that food is to satisfy hunger or relieve stress. Hence, the food ordered by participants in the interviews and surveys tended to be unhealthy, usually fast food and Asian cuisines. This is consistent with a local study in which younger participants tended to eat at fast food restaurants [49]. These options often contain a scarce amount of vegetables.

Graduate and nongraduating students were likely to be working adults (*P*<.001). These individuals experienced work stress in addition to stress from school workload compared with undergraduates. These individuals had a higher prevalence of ordering meals from MFDAs often according to the survey findings. Interviewees also expressed that they would purchase comfort food often via MFDAs, especially bubble tea, which explains the higher prevalence of drinking SSBs per day among frequent users compared with infrequent users. This quantitative finding was also evident in qualitative findings that interviewees purchased comfort food when they felt stressed. These comfort foods usually contain high amounts of sugar, and their consumption has been shown to be associated with stress relief [50].

Diet reminders have been proven to be effective in increasing uptake of healthier food choices among people who are actively dieting [51]. Diet reminders may include “My Healthy Plate,” introduced in 2014 in Singapore, which allows users to visualize whether their meal is balanced [3]. The Healthier Choice Symbol [52] in Singapore indicates which packaged food products are healthier options and which food outlets participate in the Healthier Dining Programme. These visual reminders are mostly seen in food outlets but not in MFDAs. As this study shows that the use of MFDAs is likely to be reasonably frequent for a large proportion of young adults, health policy makers could work with MFDA companies to introduce existing government-administered visual healthy choice prompts within the apps to remind young adults of the healthfulness of the meals they purchase via MFDAs.

### Cost of Food Delivery Is Trivial if Time Is Scarce

The price of food is a major barrier to using MFDAs according to the interviews. Consistent with the findings of this study, others have found that cost is the single most important determinant of food purchase among young adults [53]. If food delivery is made cheap, young adults will prefer to order from MFDAs for convenience. This was evident in the qualitative findings, in which a subgroup of interviewees from other countries (such as China and Indonesia) made comparisons with the lower cost of food delivery elsewhere. They expressed that their use frequency in these countries was much higher compared with their use in Singapore. This could be explained by Singapore’s cost of living, which has been rated as one of the highest internationally [54]. Hence, goods and services are expensive compared with those in other countries. Therefore, in Singapore’s context, it is likely that, even with discounts, food remains expensive enough to deter many young adults from using MFDAs more frequently. This is consistent with the survey findings that 58.3% (210/360) of young adults were infrequent users, which is far lower compared with 84.9% of adults in a multicountry-based study [11]. The qualitative findings showed that there are opportunities to encourage young adults to consume healthier food items. Although no study has investigated the effectiveness of discounts and cheaper food options in MFDAs to promote healthy eating, similar strategies have been used in other settings. For example, a National Discount Program in South Africa provided discounts on healthy food purchases, resulting in a substantial increase in the consumption of fruits, vegetables, and whole-grain foods and a decrease in the consumption of high-sugar, salty, fried, and processed foods [55]. Other empirical studies have shown that making healthy food items cheaper or discounted could promote healthier eating behaviors [56]. Although there are no national programs in Singapore that subsidize healthy eating, this strategy could be implemented in partnership with the government and food delivery companies. Specifically, these companies could be encouraged to offer cheaper or discounted healthy food options on their platforms.

The interviewees suggested that, although cost is central in decision-making regarding MFDA use, the need for time to finish tasks triumphed over cost. This means that young adults are willing to spend money to buy time to finish their tasks. Young adults had 2 main roles according to the survey: students and employed adults; as such, they may be facing time pressure to finish their assigned tasks, congruent with the findings of a narrative review [57]. This qualitative finding was supported by the review in that time scarcity faced by young adults is one of the determinants of consumption of takeout [57]. Consistent with the quantitative findings that lunch was the most commonly bought meal via MFDAs, if young adults were to prepare lunch, there would be opportunity costs [34], which also explains why approximately 40% (150/360, 41.7%) of the survey participants used MFDAs frequently. Hence, to avoid the use of MFDAs, public health interventions should prioritize teaching young adults time management skills, enabling them to have sufficient time to complete their tasks.

This study has several strengths. First, this mixed methods study used qualitative interviews to explain the quantitative findings in depth and provided clarifications of the phenomenon by triangulating the findings of both designs to explore why young adults reported certain patterns of MFDA use. Second, the use of the framework matrix allowed for the exploration of differences between infrequent and frequent MFDA users, which added further depth to the findings. Third, the interviewees were purposefully sampled with stratification so that the study findings could be more relevant to the demographic profile of Singaporean young adults.

### Limitations

There are several limitations to this study. First, the participants were recruited from only 1 university using convenience sampling, which may not be fully representative of all young adults in Singapore, including those who do not have access to university education. This issue was somewhat alleviated in the qualitative component, in which interviewees were purposefully selected to represent the demographic profiles of young adults more generally and ensure that the perspectives of participants exhibiting high and low MFDA use could be examined. Second, as the response rate was low, there is a possibility of nonresponse bias; nonresponders might provide differing responses from those of responders. Third, BMI reporting in the survey was subjective as survey respondents might have understated their weight to avoid feeling embarrassed, which could have resulted in information bias. Furthermore, the small sample size in the survey may have underpowered the statistical analyses, which may have contributed to the lack of significant statistical associations and the wide CIs found in this study. This limitation was mitigated by the development of the interview guide based on the quantitative results to better explain associations or differences that were not statistically significant. In the qualitative component, XCT was the only coder in this study. As such, there is a possibility that the themes might have been different if there had been a second independent coder. To alleviate this, during the later stages of analysis where latent themes were created, XCT met frequently with NAP to finalize the themes and ensure that they reflected the reality of young adults’ use of MFDAs.

### Conclusions

The study’s findings carry considerable implications for the field of public health, particularly in relation to young adults who frequently use MFDAs. These individuals are more likely to have less healthy dietary patterns, with lower vegetable consumption and higher consumption of SSBs. Future interventions could focus on promoting healthy dietary patterns by increasing vegetable consumption and reducing SSB consumption in this population. In addition, interventions could target young adults who are working or learning from home during the pandemic, promoting physical activity and healthy eating habits and reducing sedentary behavior. Moreover, the study highlights the importance of teaching basic cooking skills and promoting cooking as a habit, especially among young male individuals, to encourage healthy eating behaviors. To prevent dependence on MFDAs, it is crucial to educate young adults on effective time management skills. Cost is also a substantial consideration in food purchasing decisions, with this study suggesting that public health policies could explore ways to make healthy food options more affordable and accessible. Furthermore, this study also emphasizes the need for further research to examine the impact of COVID-19 restrictions on young adults’ dietary patterns and physical activity levels as well as evaluate the effectiveness of interventions aimed at promoting healthy behaviors during periods of social distancing and restrictions and during the *new normal* after COVID-19.

## Appendix

Multimedia Appendix 1 Quota sampling frame.

Multimedia Appendix 2 Questionnaire.

Multimedia Appendix 3 In-depth interview guide.

Multimedia Appendix 4 Univariate analysis of the associations between frequency of mobile food delivery app use and fruit and vegetable consumption, sugar-sweetened beverage consumption, and BMI.

Multimedia Appendix 5 Summary of themes, subthemes, and other examples of evidence.

## Abbreviations

aPR: adjusted prevalence ratio
CHERRIES: Checklist for Reporting Results of Internet E-Surveys
MFDA: mobile food delivery app
NCD: noncommunicable disease
NUS: National University of Singapore
REDCap: Research Electronic Data Capture
SSB: sugar-sweetened beverage
SSHSPH: Saw Swee Hock School of Public Health
WFH: work from home

## Data Availability

The data sets generated or analyzed during this study are available from the corresponding author upon reasonable request.
