# Supplementary material for: Young Adults’ Use of Mobile Food Delivery Apps and the Potential Impacts on Diet During the COVID-19 Pandemic: Mixed Methods Study
Source: JMIR Form Res. 2023 May 9;7:e38959. doi: 10.2196/38959 (PMC10173705; doi:10.2196/38959)
Supplement: Multimedia Appendix 1 [file formative_v7i1e38959_app1.pdf]

Multimedia Appendix 1

Minimum number of stratified sample based on race, sex and age (n=17).

**Race**	Chinese (n=11)						Non-Chinese (n=5)						-
**Sex**	Male (n=5)			Female (n=6)			Male (n=2)			Female (n=3)			Non-binary (Queer) (n=1)
**Age group (years)**	18-23 (n=1)	24-29 (n=2)	30-35 (n=2)	18-23 (n=2)	24-29 (n=2)	30-35 (n=2)	18-23 (n=0)	24-29 (n=1)	30-35 (n=1)	18-23 (n=1)	24-29 (n=1)	30-35 (n=1)	-

A dash "-" is inserted as there are no expected number of "Queer" individuals to be recruited.

Maximum number of stratified sample based on race, sex and age (n=25).

**Race**	Chinese (n=15)						Non-Chinese (n=8)						-
**Sex**	Male (n=7)			Female (n=8)			Male (n=4)			Female (n=4)			Non-binary (Queer) (n=2)
**Age group (years)**	18-23 (n=2)	24-29 (n=3)	30-35 (n=2)	18-23 (n=3)	24-29 (n=3)	30-35 (n=2)	18-23 (n=1)	24-29 (n=2)	30-35 (n=1)	18-23 (n=1)	24-29 (n=2)	30-35 (n=1)	-

A dash "-" is inserted as there are no expected number of "Queer" individuals to be recruited.

Number of interviewees recruited in this study stratified by race, sex and age (n=19).

**Race**	Chinese (n=13)						Non-Chinese (n=6)					
**Sex**	Male (n=6)			Female (n=7)			Male (n=3)			Female (n=3)		
**Age group (years)**	18-23 (n=2)	24-29 (n=3)	30-35 (n=1)	18-23 (n=2)	24-29 (n=3)	30-35 (n=2)	18-23 (n=1)	24-29 (n=2)	30-35 (n=0)	18-23 (n=1)	24-29 (n=2)	30-35 (n=0)
