# Supplementary material for: Young Adults’ Use of Mobile Food Delivery Apps and the Potential Impacts on Diet During the COVID-19 Pandemic: Mixed Methods Study
Source: JMIR Form Res. 2023 May 9;7:e38959. doi: 10.2196/38959 (PMC10173705; doi:10.2196/38959)
Supplement: Multimedia Appendix 2 [file formative_v7i1e38959_app2.pdf]

Multimedia Appendix 2

Mobile Food Delivery Applications Use Patterns Questions

1. How often do you do you use mobile food delivery applications to purchase food?

Never or rarely ○	Once a month ○	2-3 a month ○	Once a week ○	2-3 a week ○	4-6 a week ○	Once a day ○	2-3 a day ○	4-5 a day ○	6+ a day ○

1a*(branching if "Once a month" to "6+ a day" was selected in Question 1)*. Do you notice yourself using mobile food delivery applications more often than before this COVID-19 pandemic started?

No ○	Yes ○

1b*(branching if "Once a month" to "6+ a day" was selected in Question 1)*. Which of the following mobile food delivery applications do you usually use? (*You may choose more than one option)*

Grab □	Deliveroo □	FoodPanda □	WhyQ □	Others (please specify): ___________

1c*(branching if "Once a month" to "6+ a day" was selected in Question 1)*. Which time-period or meal do you usually purchase food via mobile food delivery applications? (*You may choose more than one option)*

Breakfast □	Lunch □	Tea Break □	Dinner □	Night Snacks □

1d*(branching if "Once a month" to "6+ a day" was selected in Question 1)*. What type of cuisine do you usually order from mobile food delivery applications? (*You may choose more than one option)*

Beverages (eg. coffee, tea, bubble tea) □	Salads □	Desserts (eg. bakery, cakes, ice cream) □	Asian-based (eg. Indian, Thai, Korean, Vietnamese, Chinese, Japanese, Singaporean, Malaysian) □	Western-based (eg. American, Italian, Mediterranean) □	Fast Food (eg. burgers, pizza, fries, fried snacks) □	Convenience food (eg microwavable food, instant noodles) □

****

Dietary Intake Questions

1. How many servings of vegetables do you eat on a typical day? (e.g, 100g raw non-leafy vegetables, ¾ mug cooked leafy or nonleafy vegetables (100g))

0 ○ 1 ○ 2 ○ 3 ○ 4 ○ 5 ○ 6 ○ >6 ○

2. How many servings of fresh fruits do you eat on a typical day? (eg, 1 small apple, orange, pear, or mango (130g), 1 wedge papaya, pineapple, or watermelon (130g))

0 ○ 1 ○ 2 ○ 3 ○ 4 ○ 5 ○ 6 ○ > 6 ○

3. How many servings of sugar-sweetened beverages do you consume on a typical day? ("Sugar-sweetened beverages" are defined as any beverages that are added with sugar, eg, 1 cup of bubble tea, 1 cup of Milo Peng, 1 can of *Coke Classic* or *Coke Zero,* 1 cup of fruit juice fresh or concentrate, 1 packet of pre-packaged Chrysanthemum tea)

0 ○ 1 ○ 2 ○ 3 ○ 4 ○ 5 ○ 6 ○ >6 ○

****

Physical Activity Questions

1. In the past 3 months, did you participate in any sports, exercise or walking during your leisure time?

No ○ Yes ○

****

Demographic Questions

1. What is your age currently? _______ years old

2. What is your gender?

Female ○ Male ○ Others (please specify): ___________

3. What is your race?

Chinese ○ Malay ○ Indian ○ Others (please specify): ___________

4. What is your height in centimetres? ________

5. What is your weight in kilograms? ________

6. What is your marital status?

Single ○ Married ○ Others (please specify): ___________

7. How many children do you have?

0 ○ 1 ○ 2 ○ Others (please specify): ___________

8. Are you currently working?

No ○ Yes ○

9. Are you an undergraduate or a graduate/nongraduating student?

Undergraduate ○ Graduate/Nongraduating ○

10. What is your current study load?

Part-Time ○ Full-Time ○

****

Interest in Qualitative Interview

1. Will you be interested in participating in qualitative interview of this research project in the future?

Yes ○ No ○ [Survey ends]

1a*(branching)*. If yes, please provide your name, phone number, and email address so that we can contact you. Thank you for your interest in the future qualitative interview.

Name: Phone number: Email address:

---THE END---
