# Supplementary material for: Young Adults’ Use of Mobile Food Delivery Apps and the Potential Impacts on Diet During the COVID-19 Pandemic: Mixed Methods Study
Source: JMIR Form Res. 2023 May 9;7:e38959. doi: 10.2196/38959 (PMC10173705; doi:10.2196/38959)
Supplement: Multimedia Appendix 3 [file formative_v7i1e38959_app3.pdf]

Multimedia Appendix 3

In-depth Interview Guide

Step 1: Introduction

- Interviewer to self-introduce
- Reiterate information in Participant Information Sheet
- If consent is given, check if there are any further questions

Hello, thank you for taking your time off to join me in this interview session. Before we start, I would like to remind you that there are no right or wrong answers in this discussion. I am interested in knowing what you think and what you have experienced. Please feel free to share your viewpoints throughout the session. If at any point of time you feel like ending the interview or if any questions make you feel uncomfortable, you can choose to end the session or not respond to the question(s). I will audio record this session and take down some field notes as I do not want to miss out any valuable information. All of your data will be coded to maintain privacy and confidentiality, and will only be shared among study team members. The interview will take around 1 hour. Do you have any questions? If not, let's get started.

Step 2: The recording will begin and the interview shall commence.

**Sample questions/probes**
Thanks for completing our survey back in January/Feb/March [i.e. whenever they completed it]. In total, we surveyed about 200 students. I'll share my screen to show you the chart. So, one of the questions was about how often students used the mobile food delivery applications or in short MFDAs, to order food. In the blue area there you see that from a total of 212 students, 127, or 2/3 of them, said they used them less than once a week. Almost 1/5 or 20% of them used the apps once a week and at least two times per week each. How do you feel about the numbers?<br><br>Probe: Are the numbers within your expectations? Why do you feel that students will not use MFDAs so frequently?/ Why do you feel that students tend to use MFDAs frequently?<br><br>Probe: In your opinion, what are some factors you think drive the frequent use of MFDAs? Which of these factors tend to drive your personal use? Why do you think so?<br><br>Probe: Which of these factors tend to drive users to purchase foods using MFDAs? Why do you think so?<br><br>Probe: As you can see from the chart, there are fewer people using the MFDAs more frequently than those who use them less than once a week. What are some of the reasons you think could have influenced them to be less frequent users of MFDAs?

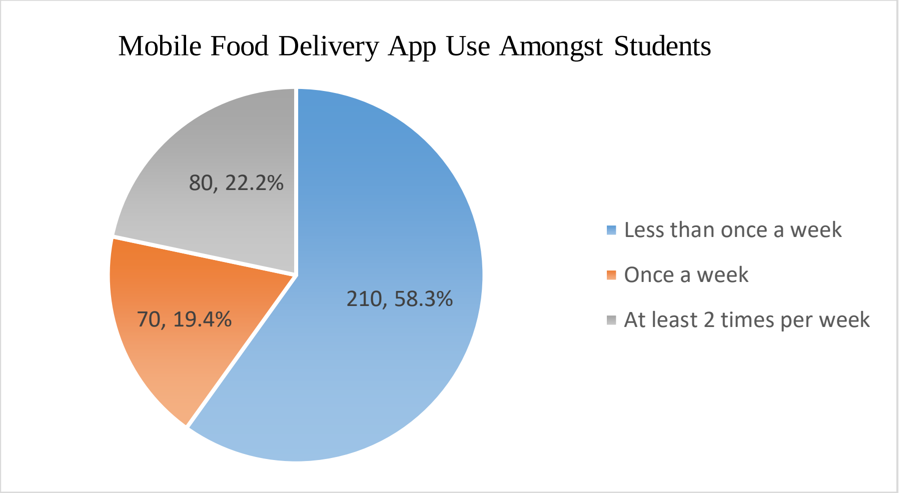

The Covid-19 pandemic has brought us to a new normal when there is less social contact between people. How do you think that pandemic has affected how young adults use MFDAs?

Probe: How has your usage of MFDAs changed during the Circuit Breaker period? What could be some possible reasons that resulted in this change?

Probe: How has your usage of MFDAs changed from pre-Covid period to the current situation?

Certain MFDAs are rather popular among the participants. What are some traits that consumers are attracted to when utilising a MFDA?

Probe: According to the survey, you tend to use Grab/Deliveroo/FoodPanda/WhyQ/other food applications [i.e. whichever they completed it]. Why?

Probe: What are the factors that entice users to certain food applications? Why are they attractive? Which are the factors that will attract you the most to use these applications?

Through the survey, it reported that the most commonly ordered meals through the app is lunch, followed by dinner. What could have possibly contributed to this?

Probe: Do you tend to order lunch more often or dinner via MFDAs?

Probe: Why do you order lunch/dinner/both lunch and dinner [i.e. whichever they completed it]. through the apps?

Probe: Why users of MFDAs are purchasing lunch and dinner? As compared to eating out, what are the enticing factors for one to buy meals via MFDAs?

The use of mobile food applications is fairly new. Do you believe that MFDAs influence the way young adults eat in anyway? Like their choice of food, meal times etc. (How?)

Probe: Do you see yourself buying more or less healthy meals through the apps? Why?/
Do you feel that young adults purchase more healthy or less healthy meals through the apps? What could be the possible reasons?

Probe: Are there specific types of foods or beverages you think young adults may be more inclined to order when using food delivery apps, compared to eating at home or physically going out to eat? Why do young adults choose them often?
Mobile food delivery applications do not require users to physically heading out to purchase their food.<br><br>Probe: How has the frequent usage of MFDAs affected your level of physical activity?/ How has physical activity level of MFDA users changed due to the convenience of food delivery?
As a student, schoolwork load tend to take up most of the hours in the day. How has your school workload affects your usage of food applications?<br><br>Probe: How has your study/school schedule affects how you use the food applications? Or perhaps the type of MFDAs that you utilise on a regular basis?
Since most of the participants are not working currently, how do you think the habits of using the food applications may change when these individuals become working adults?<br><br>Probe: Do you think your habit will change once you start working? Why? / **[For working adults]** Have you seen yourself changed your food delivery applications usage once you started working? Why?
As most of the participants surveyed are single and have no children, how will the habits of using the food applications change after they are married and have children?<br><br>Probe: Do you think your habit of using food delivery apps will change when you are married and have children? Why?/ **[For those who are married and/or have children]** Have you seen yourself changed your food delivery applications usage once you are married and/or have children? Why?
Our analysis found that individuals who use the food delivery applications more frequently, are less likely to meet the national dietary requirements of two servings of fruits and two servings of vegetables. What do you think could be the reasons behind this?<br><br>Probe: Why are frequent users of MFDAs are eating less fruits and vegetables?/ How do you find the food varieties in MFDAs? (ie Less vegetables or fruits?) Why?/Why not?
Our analysis also found that individuals who use the food delivery applications more frequently, are less likely to drink sugar-sweetened beverages or SSBs every day. Why is this so?<br><br>Probe: Why frequent users of MFDAs are drinking less SSBs?/ Why do you think infrequent users of MFDAs are drinking more SSBs? Why?/Why not?
If you were Mr Gan Kim Yong, the current Minister of Health of Singapore, will you be concerned about the impact of food delivery applications on the healthfulness of diet?

Probe: Should there be a cause of concern on how young adults use mobile food delivery apps, given that the food ordered are often less healthy? Why/why not?

Thank you for your participation. We have come to the end of the interview. Before we end, do you have any more to share regarding the topics of mobile food delivery applications?
