# Supplementary material for: Young Adults’ Use of Mobile Food Delivery Apps and the Potential Impacts on Diet During the COVID-19 Pandemic: Mixed Methods Study
Source: JMIR Form Res. 2023 May 9;7:e38959. doi: 10.2196/38959 (PMC10173705; doi:10.2196/38959)
Supplement: Multimedia Appendix 4 [file formative_v7i1e38959_app4.pdf]

Multimedia Appendix 4

Chi-square analysis of frequency of mobile food delivery app use association with diet and body mass index (N=360).

	Variables		Chi-square (df)	*P* value
**Servings of Fruits Intake per day**				
	Less than 2 (%)	At least 2 (%)		
Frequency of Mobile Food Delivery App use				
Less than once a week	154 (73.3)	56 (26.7)	0.1 (2)	.96
Once a week	52 (74.3)	18 (25.7)		
At least 2 per week	60 (75.0)	20 (25.0)		
**Servings of Vegetables Intake per day**				
	Less than 2 (%)	At least 2 (%)		
Frequency of Mobile Food Delivery App use				
Less than once a week	76 (36.2)	134 (63.8)	1.4 (2)	.50
Once a week	27 (38.6)	43 (61.4)		
At least 2 per week	35 (43.8)	45 (56.3)		
**Servings of Combined Fruits and Vegetables intake per day**				
	Less than 4 (%)	At least 4 (%)		
Frequency of Mobile Food Delivery App use				
Less than once a week	69 (32.9)	141 (67.1)	0.2 (2)	.92
Once a week	24 (34.3)	46 (65.7)		
At least 2 per week	25 (31.3)	55 (68.8)		
**Servings of Sugar Sweetened Beverages intake per day**				
	0 (%)	At least 1 (%)		
Frequency of Mobile Food Delivery App use				
Less than once a week	82 (39.1)	128 (61.0)	4.2 (2)	.12
Once a week	18 (25.7)	52 (74.3)		
At least 2 per week	27 (33.8)	53 (66.3)		
**Body Mass Index (kg/m^2)**				
	At least 23 (%)	Less than 23 (%)		
Frequency of Mobile Food Delivery App use				
Less than once a week	56 (26.7)	154 (73.3)	3.5 (2)	.18
Once a week	19 (27.1)	51 (72.9)		
At least 2 per week	30 (37.3)	50 (62.5)		
