# Supplementary material for: Young Adults’ Use of Mobile Food Delivery Apps and the Potential Impacts on Diet During the COVID-19 Pandemic: Mixed Methods Study
Source: JMIR Form Res. 2023 May 9;7:e38959. doi: 10.2196/38959 (PMC10173705; doi:10.2196/38959)
Supplement: Multimedia Appendix 5 [file formative_v7i1e38959_app5.pdf]

Multimedia Appendix 5

Summary of themes, subthemes and other examples of evidence.

Themes	Subthemes	Examples of Evidence
Theme 1: Deliberations about other sources of meals versus meals purchased via MFDAs	Subtheme 1.1: Homemade meals	"… I stay with my mother, and mothers being mothers, they just love to cook you see …" (MFDA409, frequent)<br><br>"…sometimes if I do not want to put the burden on her [my mother] like in the sense that she has to cook every day, I will suggest that we order online instead" (MFDA287, frequent).<br><br>"I'm not very good at cooking… so I … just … order food online lor" (MFDA287, frequent).<br><br>"… [I use MFDAs often]… because for dinner normally my mom would cook… so that's like less of a reason for me to use but for lunch, normally I will settle on my own then… will call in delivery" (MFDA299, frequent).
	Subtheme 1.2: Outside meals	"… if you always go out and study like go to Starbucks and study or go back to school and study, then you won't really order [MFDAs], because... like you are already outside. And you just buy food from wherever you are" (MFDA299, frequent).<br><br>"… I work …[in the] library or say Faculty of Arts and Social Sciences, or even the UTown … there are places to eat very close by, and taking a break from your study schedule means going to the cafe, means sitting with your friends once in a while… Ever since I've come to the hostel in UTown, I haven't once use the Grab App" (MFDA176, frequent).<br><br>"… presently … because I'm able to eat out and all…And school has resumed. So I tend to be outside most of the time. So there's lesser reliance on these… food delivery apps. Yeah, like even on the weekends… I have school, I can always like da bao(打包) [commonly used Chinese word for "takeaway"] home for my family. There's no

		need to… order through the app la” (MFDA234, frequent).<br><br>“… because now that the school is allowing for more physical lessons, we no longer have to stay at home to do certain activities. So since we're already outside, then maybe the mindset is that ... might as well just dine outside, instead of ...using the delivery app for ordering food” (MFDA287, frequent).
Theme 2: Convenience is vital	Subtheme 2.1: Time is of the essence	“...I have exams thrice a week. So I stay up late in school to study….” (MFDA346, frequent).<br><br>“… sometimes I have lessons also. So I have less time to cook… have other work to do. So that's why we'll order [MFDAs] for lunch… cos dinner… if my parents buy back or cook, then I wouldn't have to order” (MFDA299, frequent).<br><br>“… if I really want to focus more on my studies, I will … order from the app instead, so that I do not have to worry about ... what I want to eat next, these kind of matters .... Because I can just order from the app then continue doing my work… so it will increase my usage [of time]” (MFDA287, frequent)
	Subtheme 2.2: Staying at home	“… troublesome because if you go out, you come back, you'll need to wash your hands and change your clothes…” (MFDA465, frequent).<br><br>“… I guess is more of like variety. Because as in we always tend to buy food from [nearby]… buy from like coffee shop or food court or something like it's the same thing everyday. Or even when if it's like homecooked food and it's just feel like maybe less variety la. It adds more variety when you can get food delivery from somewhere else that you don't have to travel… I guess also for the convenience. Like for example ...I stay in Pasir Ris. Sometimes I want to order food from Tampines. But it's like too far to travel there just for like one thing, then… I'll be more

		enticed to use delivery" (MFDA299, frequent).<br><br>"I guess I'm just …really very lazy, and I just feel like ... eating something special, [so I] just order it [MFDA] lor… It's very nice. It's like somebody makes you like breakfast in bed. It's really at your doorstep. So I get this nice feeling… " (MFDA234, frequent).
Theme 3: Preference for unhealthy food ordered from MFDA most of the time	Theme 3.1: Consuming unhealthy diets is a personal satisfaction	"I'm thinking like… where is good food?... I don't think when I'm ordering, I'm wondering…is this national dietary requirements fulfilling or not? Does it have one portion of vegetables and fruits that I need to consume or not? I don't think my mind's working like that…" (MFDA176, frequent).<br><br>"… work is very tiring. Like it's a physically demanding and fast food makes you happy mah" (MFDA409, frequent).<br><br>"...I think when I'm stressed that's lesser threshold to be more… aware of [what] I am eating … so whatever guilty pleasures I have just...try to fulfill them … if I [feel] like eating … a dessert, I'll just order. The ones with drinks I just order. But it tends to be much more unhealthy things" (MFDA234, frequent).
	Theme 3.2: National dietary recommendations are non-obligatory	"… you kind of…meet the two servings of vegetables per day if you really choose consciously" (MFDA121, infrequent).<br><br>"… it's [purchasing SSBs] … like more of a habit … that every time you have a meal you need to have a drink together" (MFDA299, frequent).<br><br>"Because young adults… we are not at the point in life where we become very health conscious that we control our diets so like, we just eat to … fulfill … our desire" [MFDA287, frequent user, was seen to be laughing here].
Theme 4: Cost is king	Theme 4.1: Cost considerations	"For me, it's more worth it la, to pay for meat rather than vegetables" (MFDA38, frequent).

	when choosing vendors	"Because plain water as opposed to…Pepsi…I can easily access at my house mah... So I might as well just order something that I wouldn't have access in my house" (MFDA287, frequent).<br><br>"… I don't know why healthy food is always so expensive to purchase... Relatively very high on pricing. It would be like, why am I booked for wanting to eat healthy?" (MFDA176, frequent)<br><br>"…I know some shops. They have like slight minimum order, like probably $10 or something like that. Then I'll just order extra… like a snack or drink…to meet the minimum order" (MFDA255, frequent).
	Theme 4.2: Strategies to reduce cost	"I'll choose Grab Food because I can earn points that can be used to like reduce future costs of delivery, or … a cab ride…So you can… redeem a variety of things" (MFDA234, frequent).<br><br>"I know FoodPanda has like they give out quite a lot of vouchers like $4 off…" (MFDA287, frequent)<br><br>"… people will post online and introduce this promo code to have like a 15% discount … then I'll use [the promo code]… [on] a different app other than Grab" (MFDA299, frequent).
